# Analytical Evaluation of Stress–Strain Behavior and Reaction Mechanism of Lunar Regolith Simulant (CQU-1) Geopolymer

**DOI:** 10.3390/polym18080998

**Published:** 2026-04-20

**Authors:** Weibo Lu, Yu Shi, Xuanyi Xue, Guozhong Cheng, Honglong Li

**Affiliations:** 1 School of Civil Engineering, Chongqing University, Chongqing 400045, China; lwb386846613@163.com (W.L.);; 2 State Key Laboratory of Safety and Resilience of Civil Engineering in Mountain Area, Chongqing 400045, China

**Keywords:** lunar regolith simulant geopolymer (LRSG), compression strength, failure pattern, stress–strain behavior, reaction mechanism

## Abstract

Utilizing lunar regolith as a raw material for structural components offers significant potential for future lunar exploration. Direct manufacturing from unprocessed regolith reduces the need for specialized refining equipment compared to element extraction methods. At present, the mechanical properties of long-term alkali-activated CQU-1 lunar regolith simulant geopolymer (LRSG) columns have not been studied. To address this, forty-eight CQU-1 LRSG cylindrical specimens were prepared and tested under axial compression in this study. The effects of the curing temperature (60 °C and 80 °C), curing time (3 d, 7 d, 14 d and 28 d), and water–binder ratio (0.325 and 0.455) on the failure modes and stress–strain behavior were investigated. The alkali-activated CQU-1 LRSG achieved a maximum compressive strength of 33.89 MPa under optimal conditions. Elevated curing temperatures and extended curing times enhanced peak stress and elastic modulus while reducing peak and ultimate strains, indicating greater stiffness and brittleness. Conversely, increased water–binder ratios flattened stress–strain curves, diminishing slope and peak stress while elevating peak and ultimate strains. Based on these test results, the stress–strain model, elastic modulus model and peak strain model of alkali-activated CQU-1 LRSG were proposed. The proposed models can accurately predict the stress–strain relationship, compressive strength and ultimate strain of alkali-activated CQU-1 LRSG. The influence of curing temperature, curing time, and water–binder ratio on the performance of alkali-activated CQU-1 LRSG is also discussed in detail. This work confirms the viability of the alkali-activated CQU-1 LRSG and lunar regolith-based geopolymers for future extraterrestrial construction.

## 1. Introduction

The growing demand for Earth’s resources, coupled with advancing technology, is driving the exploration and development of extraterrestrial resources to support humanity’s sustainable development. The Moon, Earth’s only natural satellite, is a strategically prime target for deep-space exploration due to its proximity [1]. Consequently, leading space agencies have initiated major programs to explore lunar resources [2,3,4,5]. The National Aeronautics and Space Administration (NASA) aims to establish a sustainable human presence on the Moon, including the construction of a permanent lunar base. Similarly, the China National Space Administration (CNSA) is expanding its Chang’e program, with a focus on exploring the lunar south pole—a region of high interest for its potential water-ice deposits—to lay the groundwork for a future permanent base. Driven by these international efforts, lunar construction has emerged as a new research frontier that integrates aerospace engineering, materials science, and environmental science, presenting significant technical and engineering challenges. The scarcity and high cost of genuine lunar regolith make it impractical for extensive experimental use. Therefore, lunar regolith simulants, which replicate its physicochemical and mechanical properties, have become a core enabling technology for extraterrestrial exploration. Developing high-fidelity simulants is thus critical for reducing lunar exploration risks and ensuring mission success.

While numerous studies have investigated alkali-activated materials derived from terrestrial solid wastes like slags and fly ashes [6,7,8,9,10,11,12,13], their findings cannot be directly applied to lunar regolith-based polymers. Given the high cost and risk of Earth–Moon transportation, alongside increasingly ambitious and prolonged lunar missions, the goal of reducing dependence on Earth’s resources has become a consensus. Consequently, In Situ Resource Utilization (ISRU) has gained widespread recognition [14,15]. The lunar surface is subjected to prolonged meteorite impacts, cosmic radiation, and solar wind, which continuously fracture and melt surface rocks to form lunar regolith. This regolith is primarily composed of mineral fragments including feldspar, pyroxene, olivine, quartz, and ilmenite. Currently, in situ manufacturing technologies for lunar construction are broadly categorized into three groups: sintering-based [16], binder-based [17], and other additive manufacturing processes [18,19,20,21]. Various construction materials and manufacturing techniques possess distinct advantages and limitations, making them suitable for specific applications [22]. In contrast to these methods, alkali-activated lunar regolith simulant geopolymer (LRSG) offers a compelling combination of acceptable mechanical properties, excellent durability in extreme environments, low energy consumption, and a simple preparation process.

Research on alkali-activated LRSG has increased significantly in recent years. Previous studies have focused on several simulants, including JSC-1A [23], CAS-1 [24], HUST-1 [25], and BH-1 [26], but these investigations have primarily provided qualitative analysis of reaction products and matrix morphology rather than quantitative constitutive models. The mechanical properties of alkali-activated LRSG are governed by multiple factors, with activator type, dosage, and curing environment being particularly influential. Understanding how key factors govern performance evolution is critical for developing high-performance, low-cost alkali-activated LRSG materials. Such understanding is equally vital for ensuring the successful fabrication and long-term service of alkali-activated LRSG structures in the extreme lunar environment. However, current research primarily provides a qualitative analysis of reaction products and the matrix, lacking quantitative analysis across different key conditions. This gap hinders a precise evaluation of the geopolymerization process. Specifically, no existing study has systematically investigated the combined effects of curing temperature, curing time, and water–binder ratio on the full uniaxial compressive stress–strain behavior of lunar regolith simulant geopolymers, nor has a predictive model been proposed that incorporates all three parameters. Studies by Zhang et al. [24], Xiong et al. [25], and Wu et al. [26] using sodium hydroxide as an activator demonstrated that while increased alkalinity enhances aluminosilicate dissolution and geopolymer performance, excessive alkalinity degrades mechanical properties. To address these limitations, researchers have investigated milder activators, such as sodium silicate. Compounding sodium silicate with sodium hydroxide allows optimization of compressive strength by modulating the Si/Al ratio [27,28,29,30]. At an optimal Si/Al ratio, the system achieves peak compressive strength. Although sodium silicate alone can produce LRSG, the resulting mechanical strength is typically lower than that of NaOH-activated systems, leading to a scarcity of research data on its solitary use. This study employs sodium silicate as the sole activator to enable the construction of composite structures with aluminum alloy. Strong alkalis like NaOH corrode aluminum alloys via a reaction mechanism previously validated in our work (2Al + 2NaOH + 2H_2_O = 2NaAlO_2_ + 3H_2_↑) [31].

A robust constitutive model is essential for accurately characterizing the mechanical properties of lunar regolith simulants (LRSs) and serves as the foundation for efficient structural design in lunar base engineering. Gertsch prepared samples from JSC-1 lunar regolith simulant (LRS) and tested their quasi-unconfined compressive strength (QUCS), finding it was significantly influenced by water content and temperature. Liu et al. [7] systematically studied the effects of mineral composition, water content, temperature, and density on QUCS. A multiple linear regression analysis of these four factors was used to establish a predictive model for the QUCS of LRS. Atkinson et al. [23] used JSC-1A simulant to prepare samples for unconfined compressive strength (UCS) testing. Specimens (Φ25.4 mm × 63.5 mm, aspect ratio 2.5:1) were loaded at rates of 0.24–2 mm/s. Twenty tests were conducted across various conditions, including water contents of 3%, 5%, and 12% at temperatures of 196 °C and 20 °C. The maximum compressive strength of 37.15 MPa was achieved at a water content of 12%, a density of 1.85 g/cm^3^, and a temperature of 196 °C. This result differs significantly from the findings of Gertsch et al. Research on how key factors—including activator type, curing conditions, and service environment—affect the mechanical properties of alkali-activated lunar regolith simulants remains limited. Consequently, further experimental and theoretical work is required to develop a robust constitutive model. This literature review reveals a clear research gap: no existing study provides a quantitative stress–strain model for alkali-activated lunar regolith simulant geopolymers that accounts for the coupled effects of curing temperature, curing time, and water–binder ratio. The present study addresses this gap by (1) systematically testing 48 specimens under varying conditions, (2) proposing empirical models for elastic modulus and peak strain, and (3) developing a segmental stress–strain model with shape parameters correlated to key variables.

To address this research gap, a series of unconfined compressive strength tests were conducted on cylindrical LRS specimens. The experiments covered key operational parameters, including water–binder ratio, curing temperature, and curing time. The influence of these parameters on unconfined compressive strength was analyzed, and a predictive model was developed using multivariate nonlinear regression. Furthermore, microstructural and phase composition analyses were performed using X-ray diffraction (XRD). The reaction mechanism of alkali-activated LRSG was elucidated by integrating these new results with previous findings [31,32,33,34,35]. A fundamental understanding of geopolymer composition–property relationships is crucial for the rational design and optimization of tailored materials. This work provides a foundation for utilizing alkali-activated CQU-1 LRSG in lunar construction.

The novelty of this work lies in three aspects: (1) systematic investigation of the coupled effects of curing temperature, time, and water–binder ratio on the full stress–strain behavior of lunar regolith simulant geopolymer; (2) new empirical models for elastic modulus and peak strain explicitly incorporating these parameters; and (3) a segmental stress–strain model with shape parameters correlated to curing conditions. To our knowledge, no such integrated constitutive model exists for lunar regolith simulant geopolymers.

## 2. Materials and Methods

### 2.1. Materials

**Figure 1:**
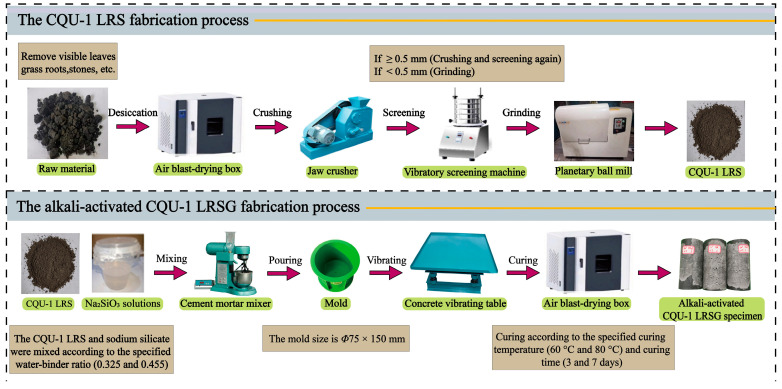
The fabrication process of CQU-1 LRS and alkali-activated CQU-1 LRSG.

**Figure 2:**
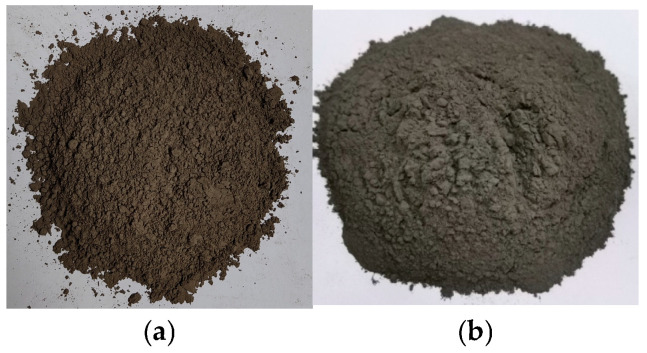
The macroscopic features of LRS raw material: (**a**) CQU-1 LRS; (**b**) HUST-1 LRS in Ref. [25].

**Figure 3:**
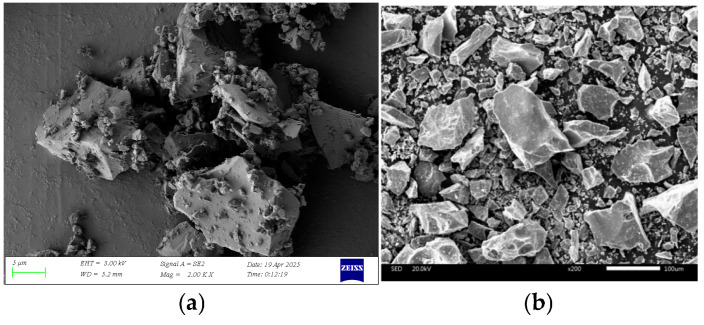
The SEM images of LRS raw material: (**a**) CQU-1 LRS; (**b**) HUST-1 LRS in Ref. [25].

The LRS raw materials used in this work originated from Jilin Province volcanic ash, sharing compositional similarity with real lunar regolith (Chang’E and Apollo 14) [2,36] and other LRSs (CAS-1 [24], HUST-1 [25], and BH-1 [26]), as detailed in Table 1. Mining involved excavating volcanic slag from open-pit deposits. Subsequent processing included crushing, drying at high temperatures, grinding, and sieving to produce the final simulant shown in Figure 1. The resulting product was designated CQU-1 LRS. Chemical composition (as shown in Table 1), morphology features (as shown in Figure 2), and scanning electron microscopy (SEM) analyses (as shown in Figure 3) confirmed the similarity between CQU-1 LRS and HUST-1 LRS [25]. Analogous validation demonstrated CQU-1 LRS’s fidelity to lunar regolith benchmarks. Additional comparative data are documented in [32].

The alkali activator used in this study was industrial-grade sodium silicate (Na_2_SiO_3_). The Na_2_SiO_3_ solution, sourced from Linyi Lvsen Chemical Co., Ltd. (Linyi, Shandong Province, China), had a silicate modulus (SiO_2_/Na_2_O) of 3.3, with 26.5% SiO_2_ and 8.3% Na_2_O (as shown in Table 2), which meets the requirements specified in Sodium Silicate for Industrial Use, China.

### 2.2. Specimen Preparation

**Figure 4:**
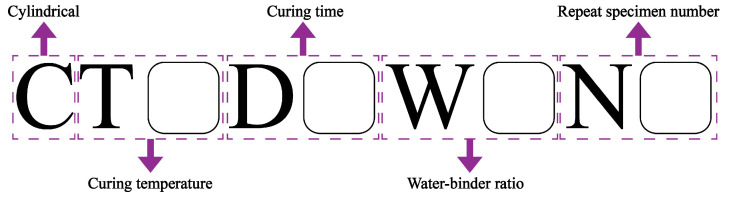
Specimen labeling.

A total of forty-eight CQU-1 LRSG cylindrical specimens were prepared in this experiment. The CQU-1 LRS was first prepared according to the method introduced in Figure 1. Then, the CQU-1 LRSG pastes were generated by mixing the CQU-1 LRS and alkali-activator solution based on the test runs with a mortar mixer. The water–binder ratios (*W*/*B*) used in this study were 0.325 and 0.455, calculated as the mass ratio of the sodium silicate solution to the CQU-1 LRS. The low-speed mixing lasted 60 s at the speed of 140 ± 5 r/min before high-speed mixing for 60 s at the speed of 285 ± 10 r/min. The CQU-1 LRSG was poured into *Φ*75 × 150 mm^3^ cylindrical molds, followed by vibration for 10 min to flatten the surface and remove entrapped air bubbles during pouring. After casting, the specimens were cured at ambient temperature (30 °C ± 1 °C) for 24 h to avoid cracks due to rapid water evaporation at high curing temperature. Then the specimens were demolded and cured in electric ovens at the corresponding curing temperatures (60 °C or 80 °C), as listed in Table 3, for durations of 3, 7, 14, or 28 days. In this study, all specimens were prepared in accordance with Chinese standards [37] and BS EN 196-1:2016 [38]. The preparation process for alkali-activated CQU-1 LRSG specimens is shown in Figure 1. All specimen labels can be seen in Figure 4.

### 2.3. Testing Methods

**Figure 5:**
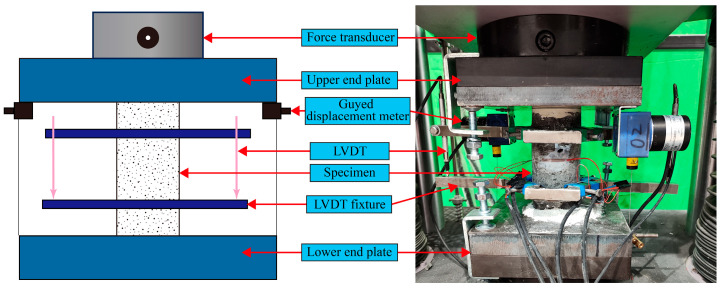
Test setup and details of instrumentation.

Axial compression tests were performed using a hydraulic servo press machine with a load capacity of 3000 kN in Chongqing University’s structural laboratory. All the test setups can be seen in Figure 5. The axial displacement of the specimen is measured by two linear variable displacement transducers (LVDTs), which were positioned 37.5 mm from the specimen’s upper and lower ends by fixtures. Two guided displacement transducers were installed between the end plates to directly measure axial deformation, compensating for system compliance errors.

**Figure 6:**
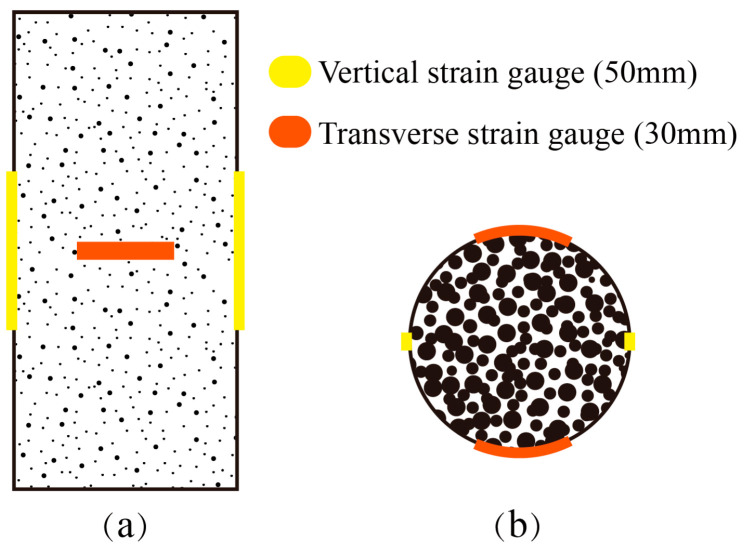
Details of strain gauge layout scheme: (**a**) front view; (**b**) vertical view.

Two guided displacement transducers were installed between the end plates to directly measure axial deformation, compensating for system compliance errors. Transverse (30 mm length) and vertical (50 mm length) strain gauges were symmetrically mounted at the specimen’s mid-height to capture axial and lateral strains during compression (Figure 6). Gypsum powder was applied to the specimen’s end surfaces to ensure uniform contact with the loading plates. The test was divided into a pre-loading stage and formal main loading stage, and the displacement control method was used to apply load to the specimen. The pre-loading proceeded at 0.2 mm/min until reaching 5% of the estimated peak load, verifying system functionality. The main loading proceeded at 0.3 mm/min, and this rate was maintained when it was close to 80% of the peak load. The loading rate was not further reduced to avoid artificial ductility; instead, the displacement rate was kept constant throughout the post-peak regime to capture the descending branch of the stress–strain curve. The testing terminated post-peak when the load decreased to 50% of the maximum or the axial strain reached 5%. The load, strain, and displacement data were synchronously recorded using a data acquisition system at a sampling frequency of 10 Hz.

## 3. Results and Discussion

### 3.1. Failure Patterns

**Figure 7:**
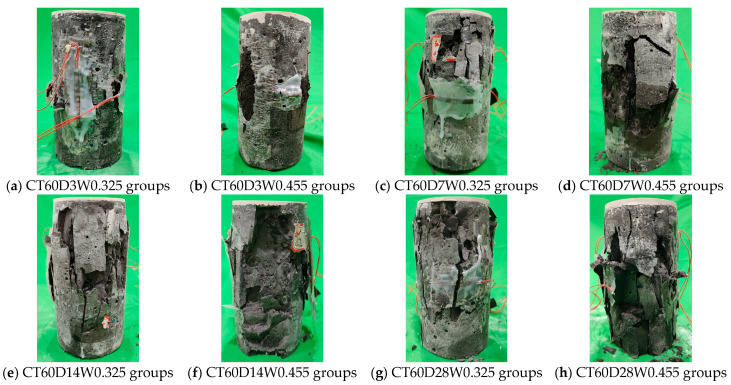
Failure pattern of alkali-activated CQU-1 LRSG cylindrical specimens with a curing temperature of 60 °C.

**Figure 8:**
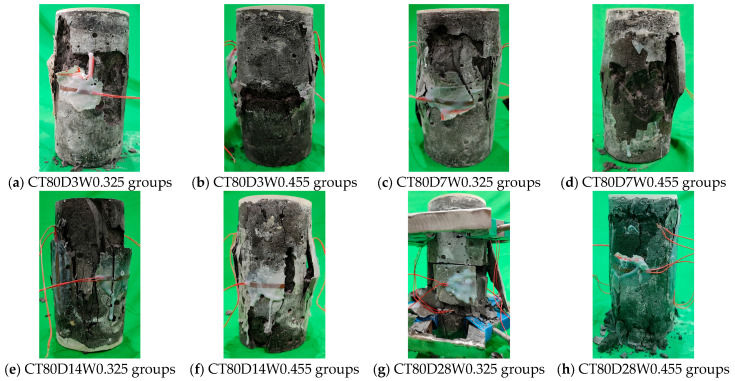
Failure pattern of alkali-activated CQU-1 LRSG cylindrical specimens with a curing temperature of 80 °C.

The typical failure mode of alkali-activated CQU-1 LRSG cylindrical specimens under unconfined uniaxial compression is shown in Figure 7 and Figure 8. The failure patterns of these specimens are analogous to those observed in conventional concrete. Upon reaching peak stress, distinct cracks propagated rapidly, causing the specimens to split into longitudinal fragments—a clear indication of brittle failure. Curing temperature and curing time significantly influence the failure mode. Specimens cured at higher temperatures (80 °C) for longer curing times (28 days) exhibited pronounced brittle failure, characterized by audible fracturing, surface spallation, a sharp post-peak load drop, and wide crack widths. This contrasts with the more ductile failure observed in short-term-cured specimens. This behavior occurs because elevated temperatures and extended curing accelerate the diffusion of reaction products into the LRS, promoting microstructural reorganization and resulting in a denser, more compact matrix. Furthermore, silicon- and aluminum-rich reaction products form a stable, dense microstructure that enhances the material’s mechanical integrity.

A comparison with our previous study using rectangular prism specimens (40 × 40 × 160 mm^3^) [31] reveals that specimen geometry influences the failure pattern. Rectangular prisms exhibited pyramidal fracture with surface spalling under compression, while the cylindrical specimens in this study failed primarily by longitudinal splitting. This difference arises from: (i) the absence of sharp corners in cylindrical specimens, which eliminates stress concentrations that can trigger premature corner cracking; (ii) the more uniform stress distribution in cylinders, promoting vertical crack propagation parallel to the loading direction; and (iii) the higher slenderness ratio of cylinders (height/diameter = 2.0) compared to rectangular prisms (height/width = 4.0), which affects the crack propagation path. The optimal curing time also increased from 7 days for rectangular prisms to 28 days for cylindrical specimens, attributed to the lower surface-area-to-volume (SA/V) ratio of cylinders (approximately 0.053 mm^−1^ vs. 0.075 mm^−1^), which reduces moisture evaporation and enables more complete geopolymerization over longer periods. Despite these geometric influences, the stress–strain behavior remains analogous between the two geometries, both exhibiting the characteristic three-stage response (elastic, elastoplastic, and failure).

### 3.2. Uniaxial Compressive Stress–Strain Curve

**Figure 9:**
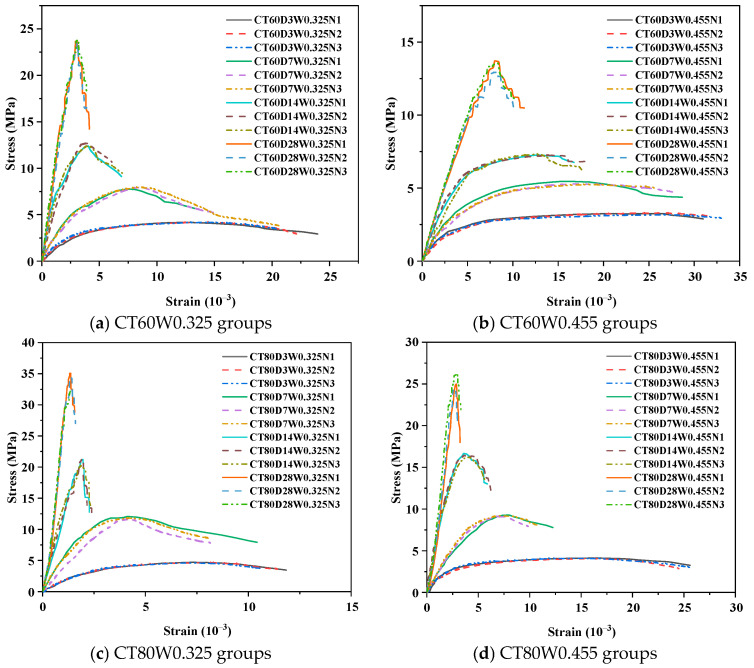
Uniaxial compressive stress–strain curve of alkali-activated CQU-1 LRSG cylindrical specimens.

The influences of curing temperatures, curing times, and water–binder ratios on the uniaxial compressive stress–strain curve of alkali-activated CQU-1 LRSG cylindrical specimens are presented in Figure 9. The stress–strain curves of alkali-activated CQU-1 LRSG resemble those of normal concrete, exhibiting ascending and descending phases. During the ascending phase, stress rises linearly with minimal strain, reflecting elastic behavior. Beyond this linear region, stress growth decelerated until peak stress was attained. Elevated curing temperatures and times increased the slope and peak stress of the ascending phase while reducing peak and ultimate strain, indicative of enhanced stiffness and brittleness. This stems from accelerated polymerization under higher temperatures and prolonged curing, which densifies the microstructure but reduces ductility, promoting brittle failure. Conversely, higher water–binder ratios flattened the stress–strain curve, reducing slope and peak stress while increasing peak and ultimate strain. This contrasts with curing-driven trends, as excessive water content caused incomplete hydration and microstructural voids, degrading mechanical performance.

### 3.3. Stress–Strain Diagram

The stress–strain behavior of the alkali-activated CQU-1 LRSG cylindrical specimen is characterized by five key parameters: peak stress (*σ*_c_p__), peak strain (*ε*_c_p__), ultimate strain (*ε*_cu_), elastic modulus (*E*_c_), and Poisson’s ratio (*υ*). Due to minimal variability within groups, reported values represent the average of three replicates per specimen group. The results are shown in Table 4 and Table 5.

#### 3.3.1. Peak Stress

**Figure 10:**
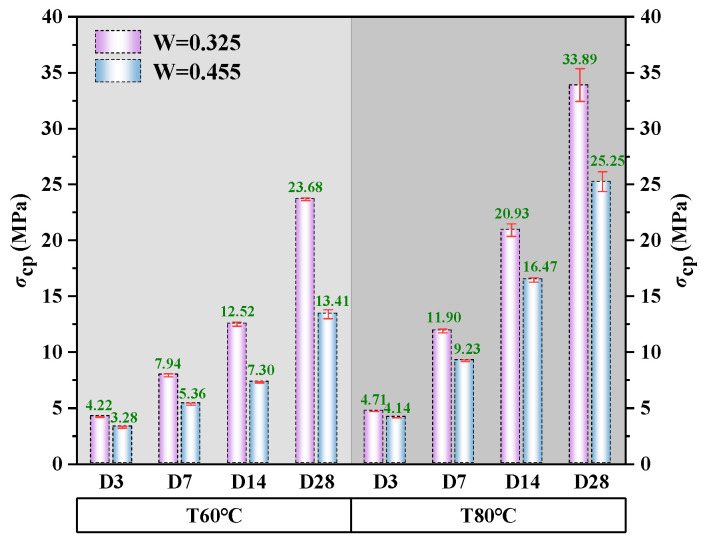
Peak stress of alkali-activated CQU-1 LRSG.

Figure 10 summarizes the average peak stress of alkali-activated CQU-1 LRSG cylindrical specimens under varying curing temperatures, curing times, and water–binder ratios. The maximum (33.89 MPa) and minimum (3.28 MPa) peak stresses occurred in the CT80D28W0.325 and CT60D3W0.455 specimens, respectively. Increasing the curing temperature from 60 °C to 80 °C raised the peak stress by 11.6% to 68.2%. Extending the curing time from 3 days to 28 days increased the peak stress by 308.8% to 619.5%. These results demonstrate that both curing temperature and time positively affect peak stress. The selected curing periods (3, 7, 14, 28 days) follow standard practice for tracking strength development in alkali-activated materials. Elevated oven curing temperatures accelerated the geopolymerization kinetics, with the most pronounced effect observed between 3 and 14 days. After 14 days, the reaction approached completion, and further strength gains were marginal. This behavior is consistent with previous studies on lunar regolith simulant geopolymers [27,28].

Moreover, regardless of the water–binder ratio, elevating the curing temperature from 60 °C to 80 °C enhanced the peak stress to varying degrees, with the most substantial improvement consistently occurring at 14 days of curing.

However, the effect of the water–cement ratio is completely opposite to that of the curing temperature and curing time. At 60 °C, increasing the water–binder ratio (0.325 to 0.455) reduced compressive strength by 22.3% at 3 days, 32.5% at 7 days, 41.7% at 14 days, and 43.4% at 28 days. At 80 °C, the same increase lowered strength by 12.1% at 3 days, 22.4% at 7 days, 21.3% at 14 days, and 25.5% at 28 days. Extended curing times and elevated curing temperatures enhanced geopolymerization, leading to a denser microstructure and higher compressive strength. In contrast, a higher water–binder ratio impeded complete geopolymerization, creating microstructural voids that degrade mechanical performance. The CQU-1 LRS content (by mass) was fixed at 75–80% of the total solid mass in all mixtures. Under optimized conditions (80 °C, 28 days, W/B = 0.325), the peak stress reached 33.89 MPa. It should be noted that the curing temperatures studied (60 °C and 80 °C) are higher than the actual lunar surface service temperatures (which range from –173 °C to 127 °C). The mechanical performance under extreme thermal cycling has not been examined in this study.

Compared with previously reported alkali-activated lunar regolith simulant geopolymers, such as JSC-1A [23], CAS-1 [24], and HUST-1 [25], the mechanical properties of CQU-1 LRSG fall within the typical range (10–60 MPa) under comparable curing conditions (60–80 °C, 7–28 days). Across all simulants, elevated curing temperatures (60–80 °C) and extended curing times (7–28 days) consistently promote N-A-S-H gel formation and microstructure densification, leading to increased compressive strength and elastic modulus but reduced ductility. Higher water–binder ratios generally reduce strength due to excess water creating voids and inhibiting complete polymerization. The failure patterns (brittle splitting or pyramidal fracture) are also similar across simulants, indicating that the fundamental failure mechanism is governed by the brittle N-A-S-H gel framework rather than simulant-specific composition. This consistency supports the applicability of the proposed constitutive models for a broader range of lunar regolith simulant geopolymers.

#### 3.3.2. Peak Strain

**Figure 11:**
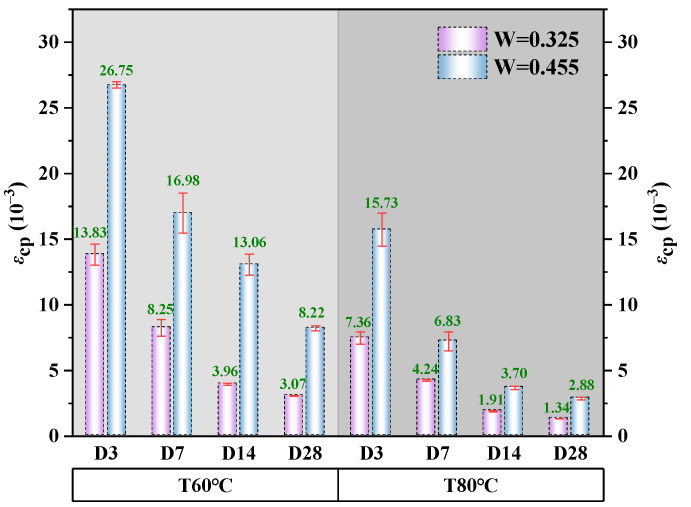
Peak strain of alkali-activated CQU-1 LRSG with different key variables.

The average peak strain average values for alkali-activated CQU-1 LRSG cylindrical specimens with different curing temperatures, curing times, and water–binder ratios are shown in Figure 11. Peak strain of alkali-activated CQU-1 LRSG cylindrical specimens decreased with higher curing temperatures and times. The maximum (26.75 × 10^−3^) and minimum (1.34 × 10^−3^) peak strains occurred in the CT60D3W0.455 and CT80D28W0.325 specimens, respectively. Increasing the curing temperature from 60 °C to 80 °C altered the peak strain from 41.2% to 71.7%. Extending the curing time from 3 to 28 days resulted in a significant reduction in peak strain. Elevated curing temperatures and extended curing times reduce the ductility of alkali-activated CQU-1 LRSG, with this effect being more pronounced under higher temperatures and longer curing times. At a curing temperature of 60 °C, increasing the water–binder ratio (0.325 to 0.455) raised peak strain by 48.3% at 3 days, 105.8% at 7 days, 229.8% at 14 days, and 167.8% at 28 days. At 80 °C, the same ratio increased peak strain by 113.7% at 3 days, 37.9% at 7 days, 48.4% at 14 days, and 114.9% at 28 days. This indicates that higher curing temperatures and times increased brittleness, while elevated water–binder ratios enhanced ductility and toughness.

#### 3.3.3. Ultimate Strain

**Figure 12:**
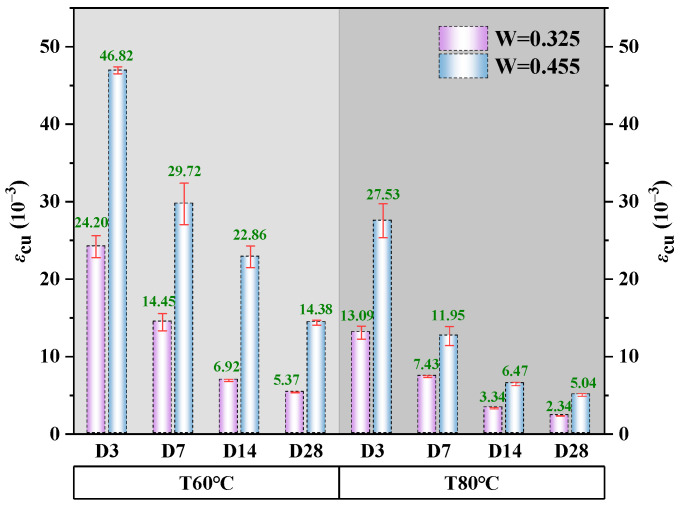
Ultimate strain of alkali-activated CQU-1 LRSG with different key variables.

The Chinese code [39] defines the ultimate strain of concrete under uniaxial compression as 50% of the strain at peak stress. When abrupt load drop-off prevents recording this value (e.g., if post-peak stress does not fall to 50% of its maximum), the ultimate strain is conservatively estimated as 1.75× the peak strain. Figure 12 summarizes the average ultimate strain of alkali-activated CQU-1 LRSG specimens across curing temperatures, curing times, and water–binder ratios. As the ultimate strain was uniformly defined as 1.75× the measured peak strain, its trends mirror those of peak strain. The CT60D3W0.455 specimens exhibited the highest ultimate strain (46.82 × 10^−3^), while CT80D28W0.325 showed the lowest (2.34 × 10^−3^). The ultimate strain decreased with elevated curing temperature (60–80 °C) and extended curing time (3–28 days), but increased with a higher water–binder ratio (0.325–0.455), mirroring the trend observed for peak strain.

#### 3.3.4. Elastic Modulus

**Figure 13:**
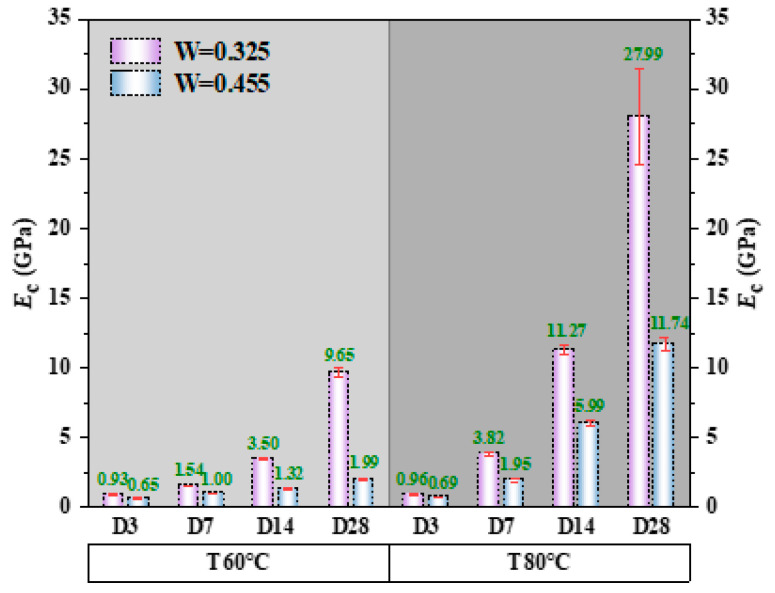
Elastic modulus of alkali-activated CQU-1 LRSG with different key variables.

Elastic modulus was derived from the initial slope of the stress–strain curve. Figure 13 shows the elastic modulus values for alkali-activated CQU-1 LRSG cylindrical specimens across curing temperatures (60–80 °C), curing times (3–28 days), and water–binder ratios (0.325–0.455). Elastic modulus ranged from 0.65 GPa to 27.99 GPa, far below conventional concrete (20–50 GPa) [40]. This shows that the alkali-activated CQU-1 LRSG cylindrical specimens have better ductility and toughness. Increasing the curing temperature from 60 °C to 80 °C raised the peak stress by 3.2% to 489.9%. Extending the curing time from 3 days to 28 days increased the peak stress by 206.2% to 2815.6%. At 60 °C, increasing the water–binder ratio (0.325 to 0.455) reduced elastic modulus by 30.1% to 79.4%. At 80 °C, the same increase lowered elastic modulus by 28.1% to 58.1%. In summary, elastic modulus trends with curing temperature, curing times, and water–binder ratio mirror those of peak stress: higher temperatures and times enhance elastic modulus, while elevated water–binder ratios degrade it.

#### 3.3.5. Poisson Ratio

**Figure 14:**
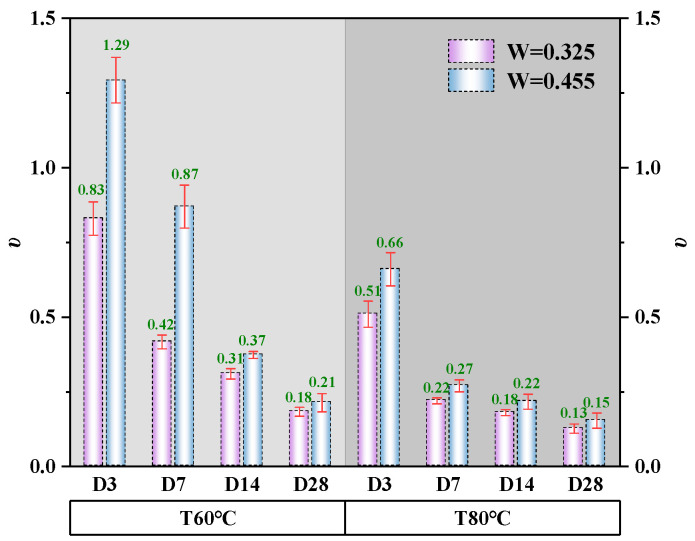
Poisson’s ratio of alkali-activated CQU-1 LRSG cylindrical specimens with different key variables.

Poisson’s ratio is defined as the ratio of transverse strain to axial strain under uniaxial loading, reflecting a material’s lateral deformation capacity. Short-age alkali-activated CQU-1 LRSG specimens exhibited Poisson’s ratios of 0.22–0.29 (Figure 14) under elevated temperatures, exceeding that of conventional concrete (≈0.20) [41]. This enhanced lateral deformability arises from effective polymerization between LRS and sodium silicate, forming a ductile geopolymer matrix. Increasing the curing temperature from 60 °C to 80 °C reduced the peak stress by 27.8% to 47.6%. Extending the curing time from 3 days to 28 days reduced the peak stress by 74.5% to 82.2%. Increasing the water–binder ratio (0.325 to 0.455) reduced elastic modulus by 30.1% to 79.4%. At 80 °C, the same increase lowered elastic modulus by 28.1% to 58.1%. However, at a curing temperature of 60 °C, increasing the water–binder ratio (0.325 to 0.455) raised Poisson’s ratio by 55.4% at 3 days, 107.1% at 7 days, 19.4% at 14 days, and 16.7% at 28 days. At 80 °C, the same ratio increased peak strain by 29.4% at 3 days, 22.7% at 7 days, 22.2% at 14 days, and 15.4% at 28 days. The enhanced lateral deformability observed in alkali-activated CQU-1 LRSG cylindrical specimens aligns with superior ductility under compression. This property warrants emphasis in structural applications, particularly for seismic-resistant designs requiring energy dissipation.

### 3.4. Stress–Strain Behavior

#### 3.4.1. Comparison of Existing Models

Classical concrete stress–strain models were applied to predict the dimensionless stress–strain behavior of alkali-activated CQU-1 LRSG cylindrical specimens. Table 6 summarizes five models and their parameters, including Guo et al. [42], Hognestad et al. [43], Chinese code [39], Carreira and Chu et al. [44] and Mukheef [45].

**Figure 15:**
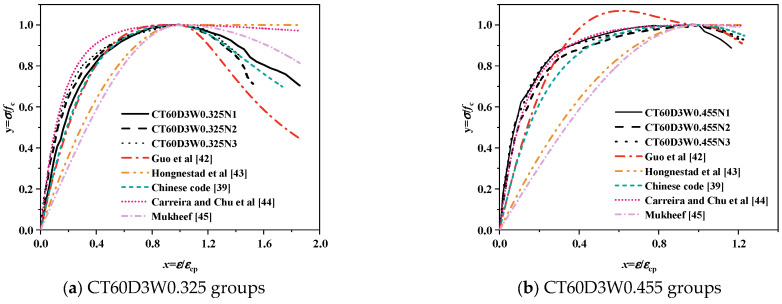

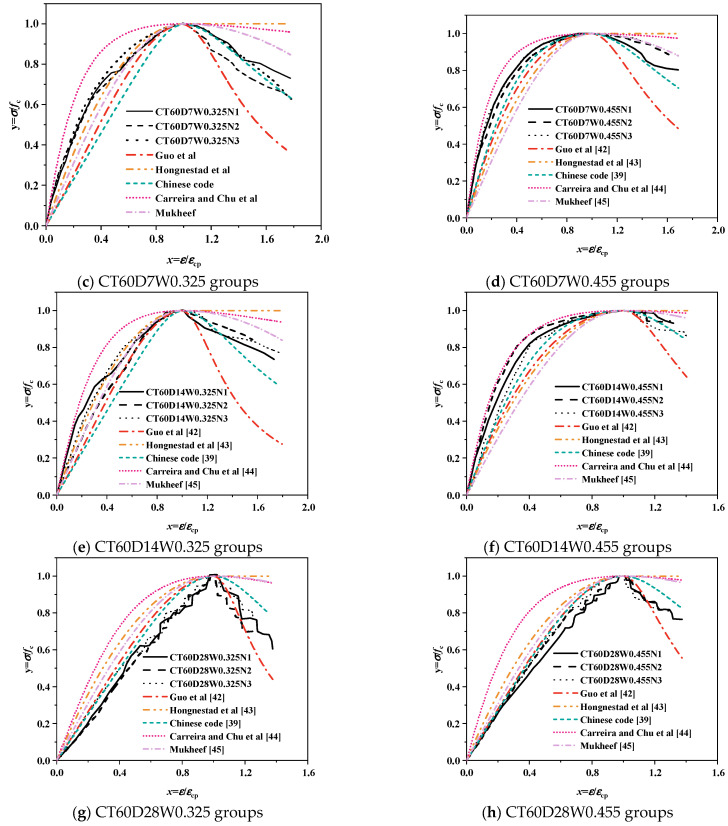
Comparison of existing stress–strain models versus test results of alkali-activated CQU-1 LRSG cylindrical specimens with a curing temperature of 60 °C [39,42,43,44,45].

**Figure 16:**
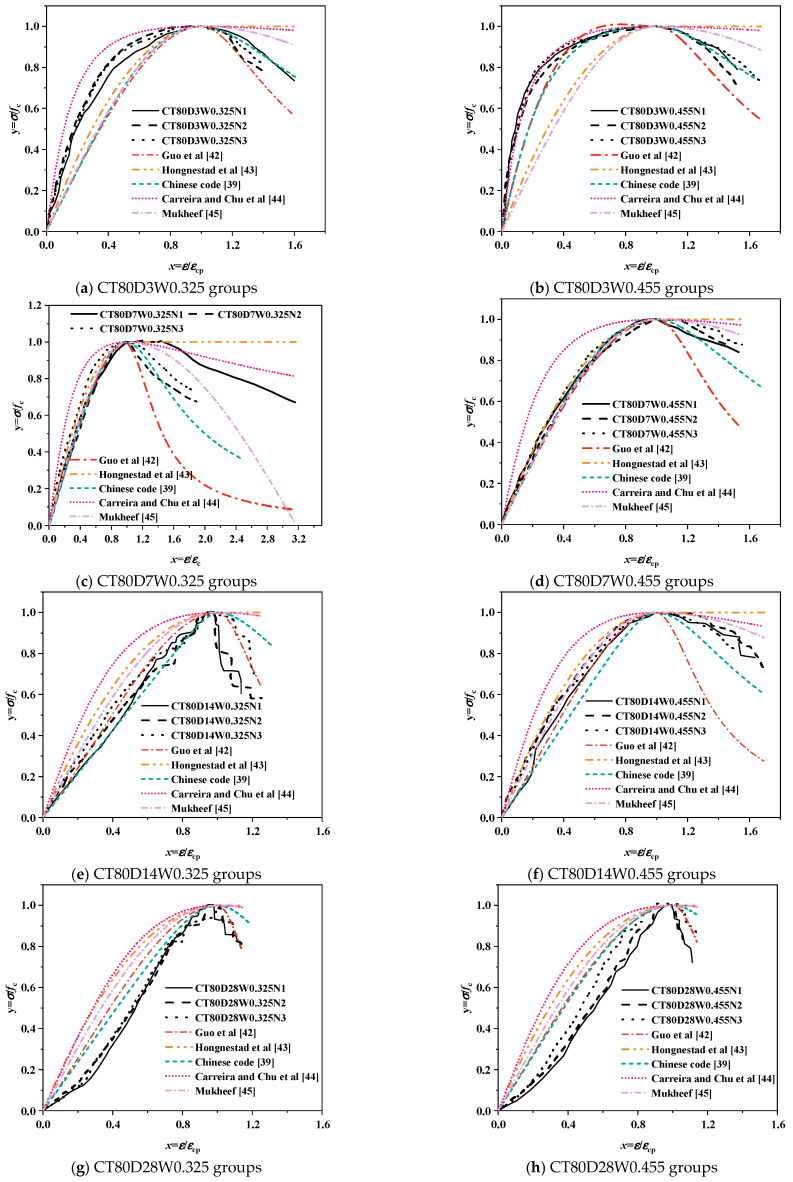
Comparison of existing stress–strain models versus test results of alkali-activated CQU-1 LRSG cylindrical specimens with a curing temperature of 80 °C [39,42,43,44,45].

Figure 15 and Figure 16 compare the experimental stress–strain curves of alkali-activated CQU-1 LRSG cylindrical specimens with predictions from existing models. Discrepancies are evident, particularly in the ascending and descending phases. The polynomial models in GB 50010-2010 [39], Guo et al. [42], and Hognestad et al. [43] use higher-degree equations to fit parameters. However, as shown in Figure 15a,f, Guo et al.’s model overestimates ascending-phase stress, producing non-physical values (σ/σ_p_ > 1), which violates material behavior constraints. Exponential [44] and trigonometric [45] models exhibit rapid stress escalation in the ascending phase but overly abrupt softening post-peak, limiting their flexibility to capture alkali-activated CQU-1 LRSG’s unique strain-softening behavior.

#### 3.4.2. Modification of Shape Parameters of Existing Models

##### Parameters β and b

To improve predictive accuracy, a segmental model integrating the ascending phase of Carreira and Chu et al.’s exponential model with the descending phase of Guo et al.’s polynomial formulation is proposed. The former aligns with experimental ascending trends, while the latter better captures post-peak softening behavior. Curing temperatures, curing times, and water–binder ratios critically influence the mechanical performance of alkali-activated CQU-1 LRSG. This study establishes relationships between key parameters (curing temperatures, curing times, water–binder ratios) and the model coefficients *β* and *b*. Regression analysis quantifies these relationships, yielding (1)–(4) for coefficients *β* and *b*, and the results are shown in Table 7.

(1) $$\left\{\begin{array}{c} Y=\frac{\beta x}{\beta -1+x^{\beta }}\;\;\;\;\;\;\;\;\;\;\;\;\;\;\;\;0\;\leq \;x\;<\;1\;\;D\;<\;14d\\ \;y=ax+\left(3-2a\right)x^{2}+\left(a-2\right)x^{3}\;\;\;\;\;0\;\leq \;x\;<\;1\;D\;\geq \;14d\\ y=\frac{x}{\;b{\left(x-1\right)}^{2}+x}\;\;\;\;\;\;\;\;\;\;\;\;\;\;\;\;\;\;\;\;\;\;\;\;\;\;\;\;x\;>\;1 \end{array}\right.$$

(2) $$\beta =0.5848\;\times \;f_{c}\;\times \;T^{-0.0075}\;\times \;D^{-0.2694}\;\times \;W^{0.3226}$$

(3) $$\;a=279.2284\;\times \;\left(\frac{E_{0}}{E_{p}}+1\right)\;\times {\;T}^{-0.0075}\;\times {\;D}^{-0.2694}\;\times \;W^{0.3226}$$

(4) $$b=2.77\;\times \;{f_{c}}^{0.795}\;\times \;T^{-0.7996}\;\times \;D^{0.2434}\;\times {\;W}^{-0.7704}$$

where *T*, *D*, and *W* represent the curing temperatures, curing times, and water–binder ratios of alkali-activated CQU-1 LRSG, respectively. The parameters *β* and *b* are linked to geopolymerization kinetics. *Β* increases with curing temperature (reflecting faster reaction rates), while *b* is related to material brittleness. The time-dependent behavior follows a power-law relationship consistent with the aging of alkali-activated materials [27]. For other simulants, material-specific constants can be recalibrated using the same methodology.

##### Elastic Modulus

**Figure 17:**
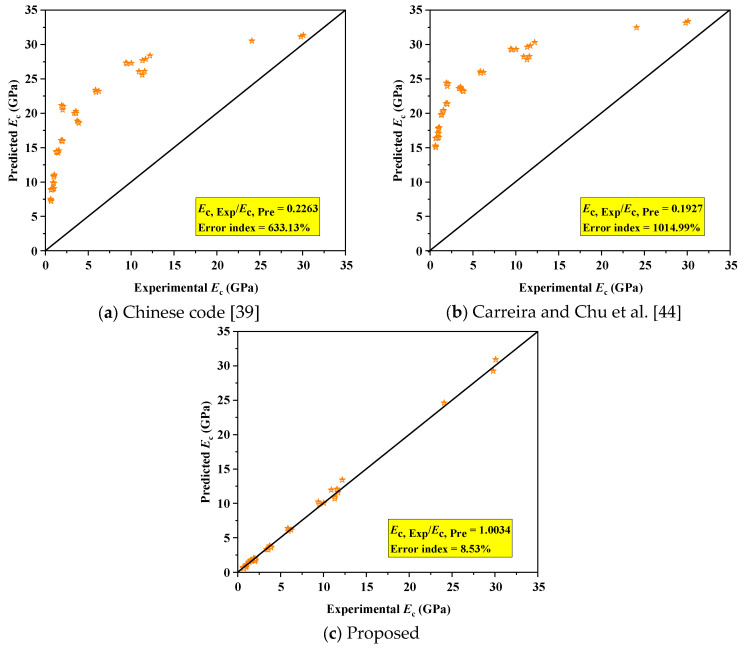
Assessment of existing and proposed models for predicting elastic modulus in alkali-activated CQU-1 LRSG. (**a**) Chinese code [39]. (**b**) Carreira and Chu et al. [44]. (**c**) Proposed.

Table 6 summarizes the elastic modulus prediction models from the Chinese code [39] and Carreira and Chu et al. [44]. The comparison results of experimental and predicted elastic modulus (proposed models and existing) are shown in Figure 17. The error index (*EI*) was used to evaluate the accuracy of the existing models, as shown in (5):

(5) $$\;EI=\sum \frac{\left|E_{c,\mathrm{Exp}.}-E_{c,\mathrm{Pre}.}\right|}{E_{c,\mathrm{Exp}.}}/n$$

where *ε*_cp,Exp._ and *ε*_cp,Pre._ are the tested and predicted elastic modulus of alkali-activated CQU-1 LRSG, respectively. The parameter *n* is the quantity of test specimens.

Both models significantly overestimate the elastic modulus of alkali-activated CQU-1 LRSG, with *EI* of 633.13% (Chinese code) and 1014.99% (Carreira and Chu et al. [44]), as shown in Figure 17a,b. This discrepancy arises because these models rely solely on compressive strength, neglecting critical factors like curing conditions and mix design. To address this, we extended these models by incorporating curing temperature, curing times, and water–binder ratio into a modified framework. Equation (6) presents the revised elastic modulus model derived via nonlinear regression analysis. Figure 17c demonstrates strong agreement between the proposed model’s predictions and experimental data. The proposed model achieves a significantly reduced *EI* (8.53%), validating its accuracy in predicting the elastic modulus of alkali-activated CQU-1 LRSG. The elastic modulus is mathematically expressed as follows:

(6) $$E_{c}\;=\;1.117\;+\;\left(0.048f_{c}-0.026W-0.262\right)f_{c}\;+\;0.202D-0.015TW$$

##### Peak Strain

**Figure 18:**
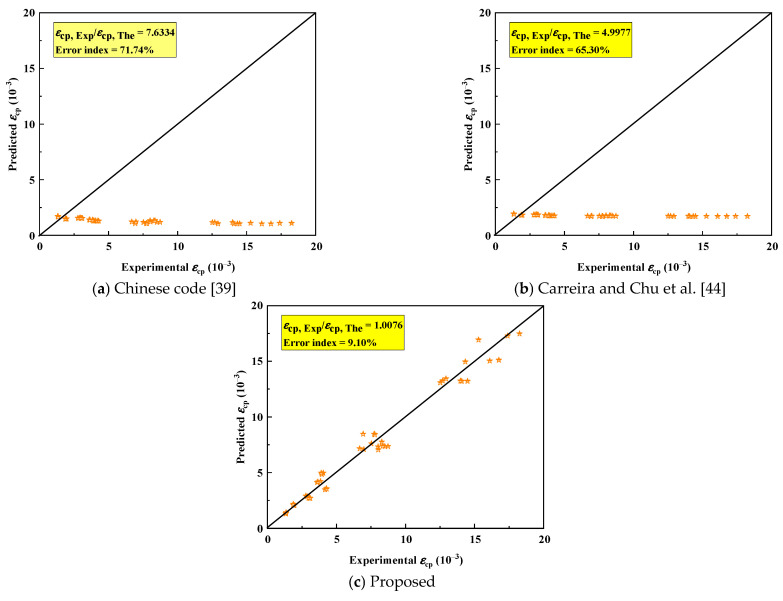
Assessment of existing and proposed models for predicting peak strain in alkali-activated CQU-1 LRSG. (**a**) Chinese code [39]. (**b**) Carreira and Chu et al. [44]. (**c**) Proposed.

The comparison results of experimental and predicted peak strain (proposed and existing models) are shown in Figure 18. Both models significantly underestimate the elastic modulus of alkali-activated CQU-1 LRSG, with *EI* of 71.74% (Chinese code) and 65.30% (Carreira et al. [44]) as shown in Figure 18a,b. Building on the elastic modulus model, we extended peak strain prediction models to incorporate curing temperature, curing time, and water–binder ratio. Equation (7) presents the modified peak strain model derived via nonlinear regression analysis. Figure 18c demonstrates strong agreement between the proposed model’s predictions and experimental results. The proposed model achieves a significantly reduced *EI* (9.10%), validating its accuracy in predicting the peak strain of alkali-activated CQU-1 LRSG.

(7) $${\;\varepsilon }_{cp}=\frac{3\;\times \;10^{4}\;\times \;D^{0.062}\;\times \;W^{1.3275}}{f_{c}\;\times \;T^{1.1868}}$$

##### Proposed Stress–Strain Model

**Figure 19:**
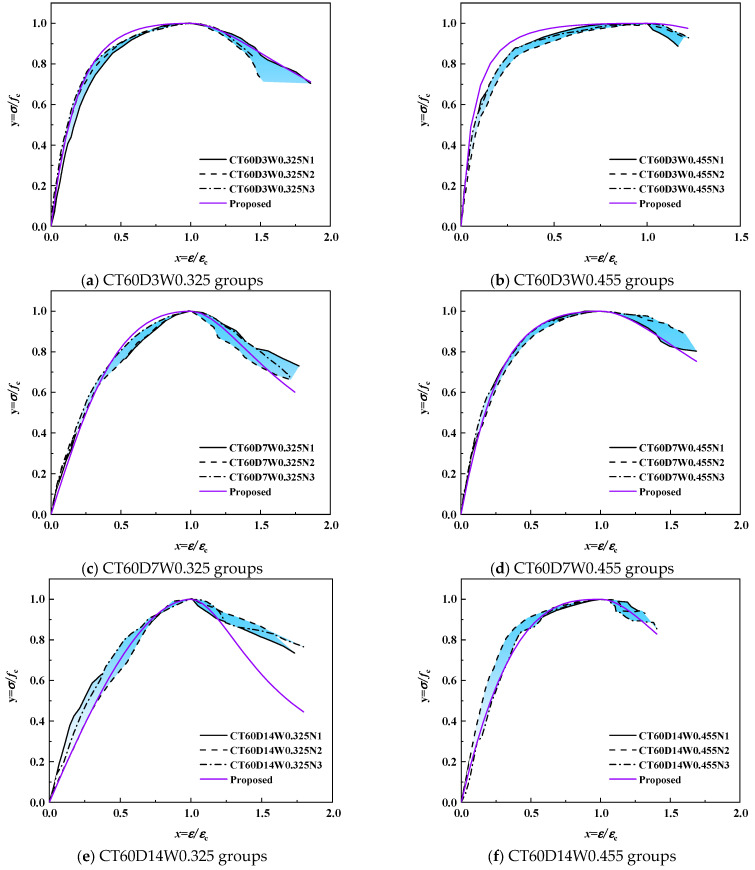

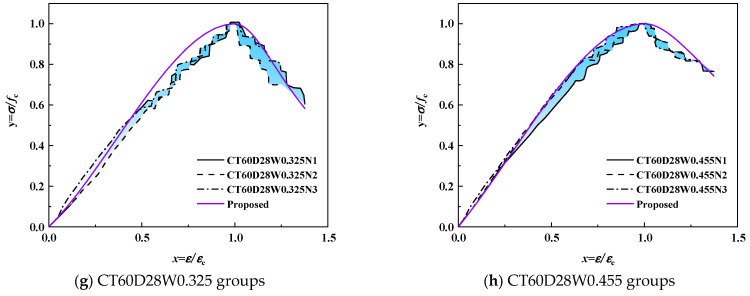
Comparison of proposed stress–strain models versus test results of alkali-activated CQU-1 LRSG cylindrical specimens with a curing temperature of 60 °C.

**Figure 20:**
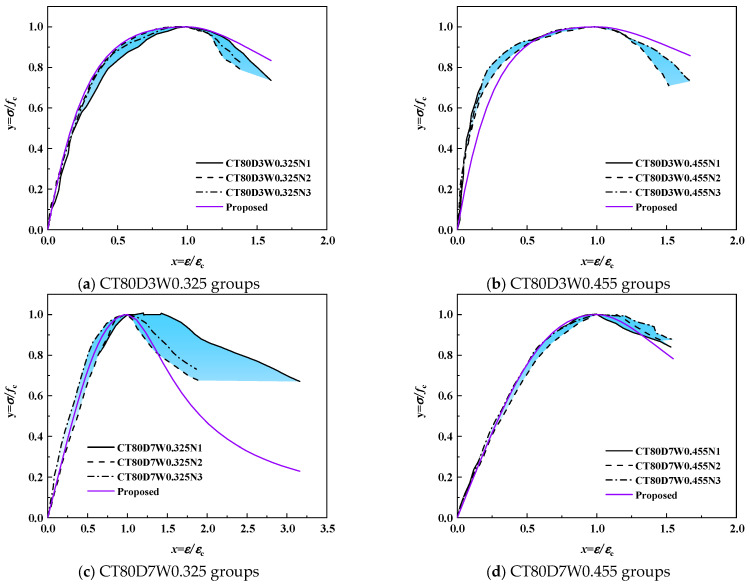

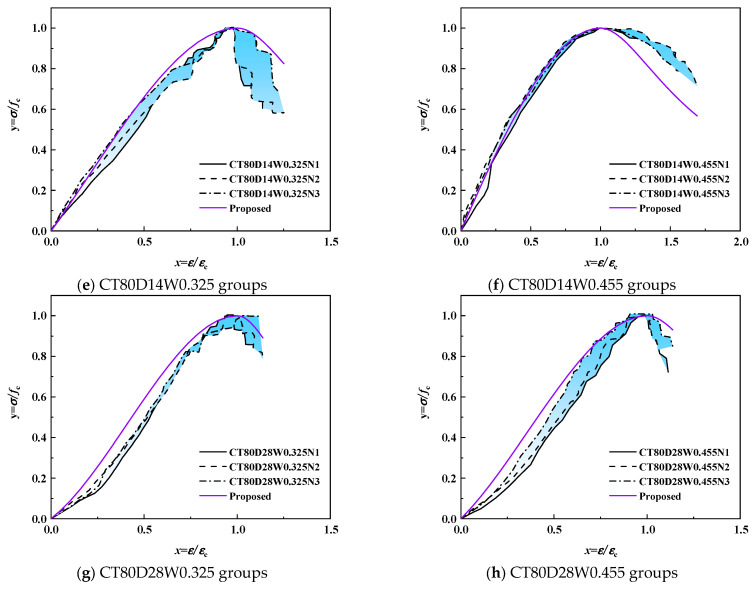
Comparison of proposed stress–strain models versus test results of alkali-activated CQU-1 LRSG cylindrical specimens with a curing temperature of 80 °C.

The proposed uniaxial compressive stress–strain model for alkali-activated CQU-1 LRSG is defined by Equation (1). Shape parameters *β*, *a*, and *b* are derived from Equations (2)–(4), while elastic modulus and peak strain are calculated via (6) and (7). Predicted stress–strain curves were validated against experimental results, as shown in Figure 19 and Figure 20. The model accurately predicts the stress–strain behavior of alkali-activated CQU-1 LRSG specimens. Thus, this segmental models effectively describes the stress–strain behavior of the unconfined alkali-activated CQU-1 LRSG subjected to static loading.

The proposed models were validated against independent experimental data from the JSC-1A [23] and HUST-1 [25] lunar regolith simulant geopolymers reported in the literature. A comprehensive review of the available literature reveals that existing studies on these simulants primarily focus on geotechnical properties or utilize sintering rather than alkali activation as the consolidation method [8,29]. Consequently, no publicly available dataset providing complete stress–strain curves with corresponding curing temperature, curing time, and water–binder ratio information exists for direct validation of the proposed constitutive models. The applicability of the proposed models to other simulants therefore remains to be verified in future studies.

## 4. Characterization of Microstructure

### 4.1. Micromorphology from SEM

**Figure 21:**
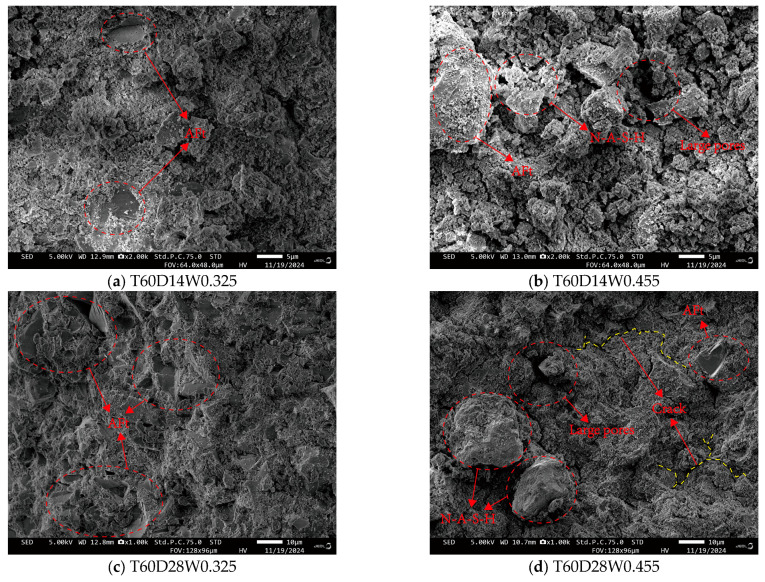
SEM of each alkali-activated CQU-1 LRSG sample with a curing temperature of 60 °C.

**Figure 22:**
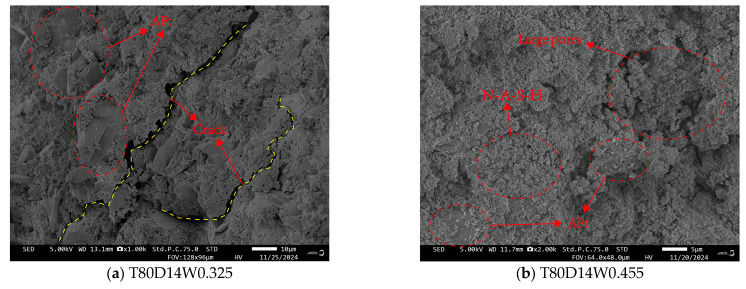

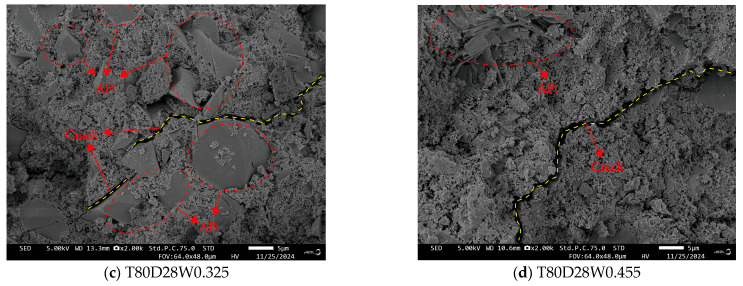
SEM of each alkali-activated CQU-1 LRSG sample with a curing temperature of 80 °C.

The SEM microstructures for specimens cured at 60 °C and 80 °C for 3 and 7 days have been reported in our previous study [31] and are not repeated here. The present study focuses on the microstructural evolution at longer curing durations (14 and 28 days), as shown in Figure 21 and Figure 22. Consistent with previous reports on early-age CQU-1 LRSG [15], shorter curing times result in an incomplete alkali activation reaction. The matrix is primarily composed of N-A-S-H gel, resulting in a loose microstructure of simulated lunar soil particles and consequently weaker mechanical properties. With extended curing, the microstructure reveals abundant plate-like or needle-like crystals, identified as Aft crystals in related studies [31,33]. This internal saturation of Aft crystals densifies the microstructure and significantly enhances mechanical performance. Microcracks were also observed, likely induced by dehydration shrinkage during high-temperature curing. Figure 21 and Figure 22 show that increasing the curing temperature from 60 °C to 80 °C significantly increased the volume of hydration products and yielded a denser microstructure, indicating that 80 °C is more effective for promoting geopolymerization. In contrast, increasing the water–binder ratio from 0.325 to 0.455 significantly increased the formation of N-A-S-H gel and other aluminosilicate gels but reduced the formation of Aft and other crystalline minerals. This suggests that an excess of these gel products may hinder further polymerization.

The microstructural evolution observed by SEM directly correlates with the mechanical properties presented in Figure 10, Figure 11, Figure 12, Figure 13 and Figure 14. Specifically, as curing time increases from 3 to 28 days, the matrix transitions from a loose particle assembly to a dense gel network, corresponding to an increase in peak stress (by 308.8–619.5%) and elastic modulus (by 206.2–2815.6%), while peak and ultimate strains decrease. This inverse relationship between densification and ductility is characteristic of brittle geopolymer materials.

### 4.2. Reaction Mechanism Analysis

This section synthesizes the present findings with prior research [32,33,34,35] to analyze the reaction mechanism of alkali-activated CQU-1 LRSG under varying curing temperatures, curing times, and water–binder ratios.

**Figure 23:**
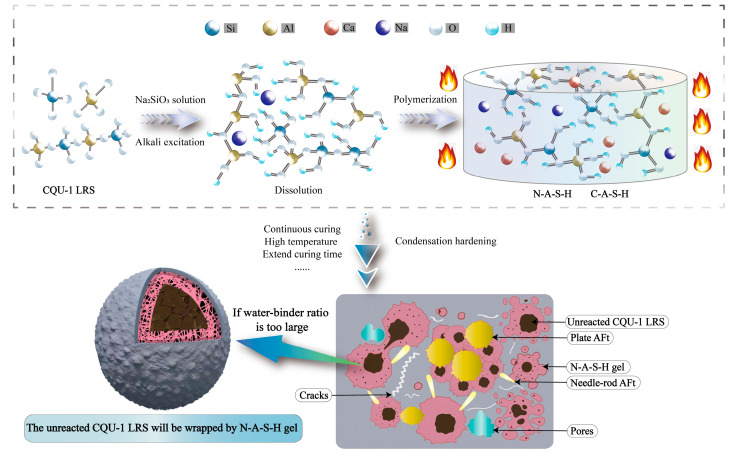
The reaction mechanism of alkali-activated CQU-1 LRSG samples.

As shown in Figure 23, the lunar regolith simulant is rich in aluminosilicate components, giving it latent reactivity. The sodium silicate (Na_2_SiO_2_) solution hydrolyzes in solution to release hydroxide ions (OH^−^), providing the necessary alkaline environment. Upon mixing, the high-pH solution attacks the simulant, breaking Si-O and Al-O bonds. The released Si, Al, and Ca ions react to form cementitious products, primarily N-A-S-H gel and AFt crystals. The sodium silicate solution provides both the alkaline environment (through hydrolysis to release OH^−^ ions) and additional silicate species that participate in the condensation reaction. The dissolved aluminosilicate species from the simulant condense with the silicate ions from the activator to form a three-dimensional N-A-S-H gel network. Concurrently, calcium ions released from the simulant react with aluminate and silicate species to form AFt (ettringite) crystals. This reaction mechanism is consistent with our SEM observations (Figure 21 and Figure 22) and with previous studies on alkali-activated lunar regolith simulants [31,32].These products bond particles together and fill pores, thereby enhancing the material’s mechanical integrity. The water–binder ratio critically influences the reaction. For instance, the high ratio of 0.455 examined here may cause an overly rapid formation of initial gel products. This can potentially hinder the long-range reorganization and densification of the geopolymer matrix, ultimately compromising the microstructure.

Three distinct features of CQU-1 LRSG are observed: (1) geopolymerization proceeds despite low amorphous glass content, enabled by fine particle size and mechanical activation; (2) AFt formation is more pronounced due to higher CaO content (8.7%); (3) the optimal curing temperature (80 °C) exceeds that of conventional geopolymers (40–60 °C), reflecting lower precursor reactivity. These features are supported by SEM evidence (Figure 21 and Figure 22) and mechanical data (Figure 10, Figure 11, Figure 12, Figure 13 and Figure 14).

The CQU-1 LRS plays a dual role in the geopolymer system: (i) as a reactive precursor, its aluminosilicate phases dissolve in the alkaline environment to form binding gels (N-A-S-H); and (ii) as a filler, unreacted particles remain embedded in the matrix, providing physical reinforcement and reducing shrinkage. This dual functionality is typical of alkali-activated materials derived from natural volcanic ashes and has been observed in other lunar regolith simulant studies [27,28,29,30].

## 5. Conclusions

The uniaxial compressive stress–strain relationship of CQU-1 LRSG cylindrical specimens was investigated, with key variables including curing temperatures, curing times, and water–binder ratios. Key findings include:

(1) Alkali-activated CQU-1 LRSG cylindrical specimens exhibited failure patterns analogous to conventional concrete. Specimens cured at lower curing temperatures (60 °C) for shorter curing times (3 days) predominantly failed through transverse midspan cracking. Under compression, they deformed laterally without audible fracturing or surface spallation, retaining structural integrity, demonstrating ductile failure. Specimens cured at higher curing temperatures (80 °C) for extended curing times (28 days) displayed brittle failure modes, marked by longitudinal and oblique cracks. Audible fracturing and surface spallation occurred during loading, consistent with brittle failure. The maximum compressive strength of alkali-activated CQU-1 LRSG cylinder specimens reached 33.89 MPa, demonstrating the potential of alkali-activated CQU-1 LRSG for lunar surface construction.

(2) The stress–strain behavior of alkali-activated CQU-1 LRSG mirrors conventional concrete, featuring elastic and post-peak phases. Elevated curing temperatures and curing times increased the slope and peak stress of the ascending phase while reducing peak and ultimate strain, indicative of enhanced stiffness and brittleness. This stems from accelerated polymerization under higher temperatures and prolonged curing, which densifies the microstructure but reduces ductility, promoting brittle failure. Conversely, higher water–binder ratios flattened the stress–strain curve, reducing slope and peak stress while increasing peak and ultimate strain. This contrasts with curing-driven trends, as excessive water content caused incomplete hydration and microstructural voids, degrading mechanical performance.

(3) Peak stress and elastic modulus increase with curing temperature (60 °C to 80 °C) and curing time (3 to 28 days), but decrease with higher water–binder ratios (0.325–0.455). Conversely, higher water–binder ratios induce incomplete hydration and microstructural voids, diminishing compressive strength and stiffness. The optimal parameter set comprises a curing temperature of 80 °C, curing time of 28 days, and water–binder ratio of 0.325.

(4) The dimensionless experimental curves were compared with predictions from classical models, including those by Guo et al. [42], Hognestad et al. [43], Chinese code [39], Carreira and Chu et al. [44] and Mukheef [45]. The Carreira et al. [44] model aligns most closely with the experimental data in the ascending phase, while the Guo et al. model better captures the descending phase. A segmental model was developed to establish functional relationships between curing temperature (*T*), curing time (*D*), water–binder ratio (*W*), and the model’s shape parameters (*β*, *b*), and elastic modulus (*E*_c_) and peak strain (*ε*_p_). Comparisons between experimental data and the proposed model confirm its effectiveness in predicting the stress–strain behavior of alkali-activated CQU-1 LRSG.

(5) A direct correlation was established: longer curing times and higher temperatures increase N-A-S-H and AFt formation, leading to higher peak stress and elastic modulus but lower peak and ultimate strains. Conversely, excess sodium silicate (*W*/*B ≥* 0.455) inhibits polymerization, resulting in porous microstructures and reduced strength.

## 6. Limitations and Future Work

Given water scarcity on the lunar surface, the optimal water–binder ratio of 0.325 identified in this study represents a trade-off between mechanical performance and resource utilization. Future work should explore water recovery from polar ice deposits [14] or the use of near-zero-water activators [3] to reduce dependence on Earth-supplied water. The higher water–binder ratio (0.455) is not recommended for lunar applications due to its significantly lower strength, despite its higher ductility.

The present study was conducted under terrestrial conditions (ambient pressure, Earth gravity, controlled laboratory temperature). Actual lunar conditions involve ultra-high vacuum (10^−14^ to 10^−12^ Pa), reduced gravity (1/6 g), and extreme temperature fluctuations (−173 °C to 127 °C). These factors may affect the geopolymerization reaction (e.g., water evaporation under vacuum, altered reaction kinetics) and the long-term durability of the material. Therefore, the results presented here should be considered as a baseline for terrestrial-simulant testing. Future work must validate these findings under simulated lunar environmental conditions, including vacuum chambers, thermal cycling, and reduced-gravity parabolic flights. Until such validation, direct extrapolation to lunar construction should be made with caution.

## Figures and Tables

**Table 1:** Chemical compositions of lunar regolith simulants and real lunar regolith (% by weight).

Oxides	SiO_2_	TiO_2_	Al_2_O_3_	Fe_2_O_3_	CaO	MgO	Na_2_O	K_2_O	P_2_O_5_
CQU-1	45.31	2.80	15.01	15.67	8.34	3.41	4.50	3.33	0.65
Chang’e-5 [2]	42.20	5.00	10.80	22.50	11.00	6.48	0.26	0.19	0.23
CAS-1 [24]	49.24	1.91	15.80	11.47	7.25	8.72	3.08	1.03	0.30
HUST-1 [25]	48.23	2.96	18.29	11.19	7.89	4.41	3.70	2.15	0.50
BH-1 [26]	43.30	2.90	16.50	16.70	8.80	3.00	3.80	3.30	0.70
Apollo 14 [36]	48.10	1.70	17.40	10.40	10.70	9.40	0.70	0.55	0.51

**Table 2:** Technical parameters of sodium silicate (Na_2_SiO_2_) solution.

Material	SiO_2_ (%)	Na_2_O (%)	Densities (g/mL)	Modulus	Baume Degrees
Na_2_SiO_2_	26.5	8.3	1.368~1.381	3.20~3.40	39.0~40.0

**Table 3:** Considered parameters of alkali-activated CQU-1 LRSG specimens.

Specimen ID	Ball Milling Time (min)	Modulus	LRS (g)	Na_2_SiO_3_ (g)	Curing Temperature (°C)	Curing Time (d)	Water–Binder Ratio
CT60D3W0.325N1, 2, 3	30	3.3	100	33.33	60	3	0.325
CT60D3W0.455N1, 2, 3				41.17			0.455
CT60D7W0.325N1, 2, 3				33.33		7	0.325
CT60D7W0.455N1, 2, 3				41.17			0.455
CT60D14W0.325N1, 2, 3				33.33		14	0.325
CT60D14W0.455N1, 2, 3				41.17			0.455
CT60D28W0.325N1, 2, 3				33.33		28	0.325
CT60D28W0.455N1, 2, 3				41.17			0.455
CT80D3W0.325N1, 2, 3				33.33	80	3	0.325
CT80D3W0.455N1, 2, 3				41.17			0.455
CT80D7W0.325N1, 2, 3				33.33		7	0.325
CT80D7W0.455N1, 2, 3				41.17			0.455
CT80D14W0.325N1, 2, 3				33.33		14	0.325
CT80D14W0.455N1, 2, 3				41.17			0.455
CT80D28W0.325N1, 2, 3				33.33		28	0.325
CT80D28W0.455N1, 2, 3				41.17			0.455

**Table 4:** Characteristic indices of stress–strain curves with a curing temperature of 60 °C.

Specimen ID	*σ*_c_p__ (Mpa)	Avg. (Mpa)	*ε*_c_p__ (10^−3^)	Avg. (10^−3^)	*ε*_cu_ (10^−3^)	Avg. (10^−3^)	*E*_c_ (Gpa)	Avg. (GPa)	*υ*	Avg.
CT60D3W0.325N1	4.17	4.22	12.93	13.83	22.63	24.20	0.91	0.93	0.88	0.83
CT60D3W0.325N2	4.24		14.49		25.36		0.88		0.84	
CT60D3W0.325N3	4.24		14.07		24.62		1.01		0.77	
CT60D3W0.455N1	3.26	3.28	26.66	26.75	46.66	46.82	0.67	0.65	1.31	1.29
CT60D3W0.455N2	3.36		26.57		46.50		0.62		1.21	
CT60D3W0.455N3	3.21		27.03		47.30		0.66		1.36	
CT60D7W0.325N1	7.77	7.94	7.53	8.25	13.18	14.45	1.49	1.54	0.43	0.42
CT60D7W0.325N2	8.02		8.73		15.28		1.58		0.39	
CT60D7W0.325N3	8.02		8.50		14.88		1.55		0.43	
CT60D7W0.455N1	5.45	5.36	15.29	16.98	26.76	29.72	1.06	1.00	0.81	0.87
CT60D7W0.455N2	5.34		17.40		30.45		0.97		0.95	
CT60D7W0.455N3	5.28		18.26		31.96		0.97		0.85	
CT60D14W0.325N1	12.42	12.52	4.04	3.96	7.07	6.92	3.37	3.50	0.29	0.31
CT60D14W0.325N2	12.73		3.94		6.90		3.56		0.32	
CT60D14W0.325N3	12.40		3.89		6.81		3.57		0.32	
CT60D14W0.455N1	7.27	7.30	12.69	13.06	22.21	22.86	1.37	1.32	0.36	0.37
CT60D14W0.455N2	7.28		13.98		24.47		1.29		0.38	
CT60D14W0.455N3	7.36		12.52		21.91		1.31		0.38	
CT60D28W0.325N1	23.69	23.68	3.07	3.07	5.37	5.37	10.03	9.65	0.17	0.18
CT60D28W0.325N2	23.56		3.04		5.32		9.51		0.20	
CT60D28W0.325N3	23.79		3.10		5.43		9.41		0.18	
CT60D28W0.455N1	13.71	13.41	8.01	8.22	14.02	14.38	1.89	1.99	0.18	0.21
CT60D28W0.455N2	12.96		8.28		14.49		2.01		0.24	
CT60D28W0.455N3	13.56		8.36		14.63		2.09		0.22	

**Table 5:** Characteristic indices of stress–strain curves with a curing temperature of 80 °C.

Specimen ID	*σ*_c_p__ (Mpa)	Avg. (Mpa)	*ε*_c_p__ (10^−3^)	Avg. (10^−3^)	*ε*_cu_ (10^−3^)	Avg. (10^−3^)	*E*_c_ (Gpa)	Avg. (GPa)	*υ*	Avg.
CT80D3W0.325N1	4.70	4.71	7.73	7.36	13.53	13.09	1.00	0.96	0.56	0.51
CT80D3W0.325N2	4.73		7.78		13.62		0.91		0.48	
CT80D3W0.325N3	4.70		6.93		12.13		0.96		0.49	
CT80D3W0.455N1	4.12	4.14	16.76	15.73	29.33	27.53	0.66	0.69	0.61	0.66
CT80D3W0.455N2	4.14		16.10		28.18		0.69		0.65	
CT80D3W0.455N3	4.16		14.33		25.08		0.72		0.72	
CT80D7W0.325N1	12.08	11.90	4.17	4.24	7.30	7.43	3.71	3.82	0.23	0.22
CT80D7W0.325N2	11.74		4.27		7.47		3.90		0.21	
CT80D7W0.325N3	11.89		4.29		7.51		3.86		0.22	
CT80D7W0.455N1	9.29	9.23	8.03	6.83	14.05	11.95	1.86	1.95	0.27	0.27
CT80D7W0.455N2	9.16		6.68		11.69		1.98		0.29	
CT80D7W0.455N3	9.25		6.98		12.21		2.00		0.25	
CT80D14W0.325N1	21.21	20.93	1.96	1.91	3.43	3.34	10.92	11.27	0.17	0.18
CT80D14W0.325N2	21.30		1.89		3.31		11.58		0.18	
CT80D14W0.325N3	20.28		1.87		3.27		11.31		0.19	
CT80D14W0.455N1	16.67	16.47	3.60	3.70	6.30	6.47	5.85	5.99	0.22	0.22
CT80D14W0.455N2	16.46		3.65		6.39		6.23		0.19	
CT80D14W0.455N3	16.28		3.84		6.72		5.90		0.24	
CT80D28W0.325N1	35.06	33.89	1.32	1.34	2.31	2.34	30.09	27.99	0.13	0.13
CT80D28W0.325N2	34.35		1.34		2.35		29.80		0.11	
CT80D28W0.325N3	32.26		1.35		2.36		24.08		0.14	
CT80D28W0.455N1	24.99	25.25	2.88	2.88	5.04	5.04	11.69	11.74	0.13	0.15
CT80D28W0.455N2	24.53		2.78		4.87		11.33		0.15	
CT80D28W0.455N3	26.23		2.98		5.22		12.20		0.18	

**Table 6:** Existing stress–strain models for classical concrete.

Ref.	Stress–Strain Model	Characteristic	Parameters
Guo et al. [42]	$\;y=ax+\left(3-2a\right)x^{2}+\left(a-2\right)x^{3}\;0\;\leq \;x\;<\;1$ $y=\frac{x}{b{\left(x-1\right)}^{2}+x}\;x\;>\;1$	-	*a* is the ratio of the initial elastic modulus to the secant elastic modulus; *b* is determined based on the concrete strength grade and constraint method.
Hognestad et al. [43]	$\;y=2x-x^{2}\;0\;\leq \;x\;<\;1$ y=1 x > 1	-	-
Chinese code [39]	$\;y=nx/\left(n-1+x^{n}\right)\;0\;\leq \;x\;<\;1$ $y=\;x/\left[\alpha_{c}{\left(x-1\right)}^{2\;}+x\right]\;x\;>\;1$	${\;E}_{c}=\frac{10^{5}}{2.2+34.7/f_{c}}$ ${\;\varepsilon }_{\mathrm{cp}}=\left(172\sqrt{\sigma_{\mathrm{cp}}\;}+700\right)\times 10^{-6}$	$\;n=1/\left(1-E_{\sec}/E_{c}\right)$ $\alpha_{c}=0.157{f_{c}}^{0.785}-0.905$
Carreira and Chu et al. [44]	$\;y=\frac{\beta x}{\beta -1+x^{\beta }}$	${\;E}_{c}=10200{\left(f_{c}\right)}^{1/3}$ ${\;\varepsilon }_{\mathrm{cp}}=\left(0.71f_{c}+168\right)\;\times \;10^{-5}$	$\;\beta =1/\left(1-\frac{f_{c}}{{\;\varepsilon }_{c}{\;E}_{c}}\right)\;\;$
Mukheef [45]	$\;y=\sin\left(\frac{\pi }{2}x\right)$	-	-

Note: $x\;=\;\varepsilon /\varepsilon_{p}$, $y\;=\;\sigma /\sigma_{p}\;=\;\;\sigma /f_{c}\;$.

**Table 7:** Parameters *β* and *b* for the proposed model.

Specimen ID	β	*a*	*b*	Specimen ID	β	*a*	*b*
CT60D3W0.325	1.238	-	1.023	CT80D3W0.325	1.379	-	0.887
CT60D3W0.455	1.073	-	0.646	CT80D3W0.455	1.351	-	0.617
CT60D7W0.325	1.855	-	2.078	CT80D7W0.325	2.774	-	2.277
CT60D7W0.455	1.395	-	1.173	CT80D7W0.455	2.398	-	1.436
CT60D14W0.325	-	1.622	3.534	CT80D14W0.325	-	1.282	4.224
CT60D14W0.455	-	2.928	1.776	CT80D14W0.455	-	1.585	2.694
CT60D28W0.325	-	0.568	6.943	CT80D28W0.325	-	0.712	7.335
CT60D28W0.455	-	0.867	3.409	CT80D28W0.455	-	0.915	4.480

## Data Availability

The raw data supporting the conclusions of this article will be made available by the authors on request.

## References

[1] Degtyarev A.V., Lobanov L.M., Kushnar’ov A.P., Baranov I.Y., Volkov V.S., Perepichay A.O., Korotenko V.V., Volkova O.A., Osinovyy G.G., Lysenko Y.A. (2020). On possibilities for development of the common-sense concept of habitats beyond the Earth. ACTA Astronaut..

[2] Li C., Hu H., Yang M.F., Pei Z.Y., Zhou Q., Ren X., Liu B., Liu D., Zeng X., Zhang G. (2022). Characteristics of the lunar samples returned by the Chang’E-5 mission. Natl. Sci. Rev..

[3] Toklu Y.C., Akpinar P. (2022). Lunar soils, simulants and lunar construction materials: An overview. Adv. Space Res..

[4] Creech S., Guidi J., Elburn D. (2022). IEEE Artemis: An Overview of NASA’s Activities to Return Humans to the Moon. 2022 IEEE Aerospace Conference (AERO).

[5] Zheng X.W., Zhao C., Sun X.Y., Dong W.W. (2024). Lunar Regolith Geopolymer Concrete for In-Situ Construction of Lunar Bases: A Review. Polymers.

[6] Hou X.Y., Ding T.X., Chen T., Liu Y.M., Li M., Deng Z.Q. (2019). Constitutive properties of irregularly shaped lunar soil simulant particles. Powder Technol..

[7] Wang S., Jiang M., Zhao T., Shi A. (2024). Analyzing strain localization of Chang’E-5 lunar regolith through discrete element analysis. Powder Technol..

[8] Sun Z.Q., Vollpracht A. (2018). Isothermal calorimetry and in-situ XRD study of the NaOH activated fly ash, metakaolin and slag. Cem. Concr. Res..

[9] Gebregziabiher B.S., Thomas R., Peethamparan S. (2015). Very early-age reaction kinetics and microstructural development in alkali-activated slag. Cem. Concr. Compos..

[10] Ma H., Wu C. (2022). Mechanical and microstructural properties of alkali-activated fly ash-slag material under sustained moderate temperature effect. Cem. Concr. Compos..

[11] Chen L.C., Wang T., Li F., Zhou S.Q. (2024). Preparation of geopolymer for in-situ pavement construction on the moon utilizing minimal additives and human urine in lunar regolith simulant. Front. Mater..

[12] Geng Z., Zhang L., Pan H., She W., Zhou C., Zhou H., Yu Z., Xu Z. (2023). In-situ solidification of alkali-activated lunar regolith: Insights into the chemical and physical origins. J. Clean. Prod..

[13] Peng B., Hay R., Celik K. (2023). 3D shape analysis of lunar regolith simulants. Powder Technol..

[14] Sanders G.B., Larson W.E. (2013). Progress Made in Lunar In Situ Resource Utilization under NASA’s Exploration Technology and Development Program. J. Aerosp. Eng..

[15] Sun X., Zhang R., Li X., Zou M., Zhang H. (2026). Calibration of simulation parameters for in-situ excavation of lunar highland simulant. Powder Technol..

[16] Meurisse A., Beltzung J.C., Kolbe M., Cowley A., Sperl M. (2017). Influence of Mineral Composition on Sintering Lunar Regolith. J. Aerosp. Eng..

[17] Lin T.D., Skaar S.B., OGallagher J.J. (1997). Proposed remote-control, solar-powered concrete production experiment on the moon. J. Aerosp. Eng..

[18] Geng Z.F., Pan H., Zuo W.Q., She W. (2022). Functionally graded lightweight cement-based composites with outstanding mechanical performances via additive manufacturing. Addit. Manuf..

[19] Geng Z.F., Wu P.P., Pan H., Zheng Q., Zuo W.Q., Zhang W.H., She W. (2022). Robust layer interface in cement additive manufacturing via silicate penetration and precipitation. Mater. Des..

[20] Hu Z.J., Shi T., Cen M.Q., Wang J.M., Zhao X.Y., Zeng C., Zhou Y., Fan Y.J., Liu Y.M., Zhao Z.F. (2022). Research progress on lunar and Martian concrete. Constr. Build. Mater..

[21] Ngo T.D., Kashani A., Imbalzano G., Nguyen K.T.Q., Hui D. (2018). Additive manufacturing (3D printing): A review of materials, methods, applications and challenges. Compos. Part B Eng..

[22] Assi L.N., Carter K., Deaver E., Ziehl P. (2020). Review of availability of source materials for geopolymer/sustainable concrete. J. Clean. Prod..

[23] Xiao J.X., Tang J.Y., Lu Z.X., Chi C., Sun M., Zhang W.W., Chi R.Q., Jiang S.Y. (2023). Dynamic behavior and modeling of icy lunar regolith subjected to dynamic loading. ACTA Astronaut..

[24] Zheng Y., Wang S., Ouyang Z., Zou Y., Liu J., Li C., Li X., Feng J. (2009). CAS-1 lunar soil simulant. Adv. Space Res..

[25] Han W., Ding L., Cai L., Zhu J., Luo H., Tang T. (2022). Sintering of HUST-1 lunar regolith simulant. Constr. Build. Mater..

[26] Zhou S., Lu C., Zhu X., Li F. (2021). Preparation and Characterization of High-Strength Geopolymer Based on BH-1 Lunar Soil Simulant with Low Alkali Content. Engineering.

[27] Xiong G.Y., Guo X.L., Yuan S.T., Xia M., Wang Z.H. (2022). The mechanical and structural properties of lunar regolith simulant based geopolymer under extreme temperature environment on the moon through experimental and simulation methods. Constr. Build. Mater..

[28] Zhou S.Q., Zhu X.Y., Lu C.H., Li F. (2022). Synthesis and characterization of geopolymer from lunar regolith simulant based on natural volcanic scoria. Chin. J. Aeronaut..

[29] Montes C., Broussard K., Gongre M., Simicevic N., Mejia J., Tham J., Allouche E., Davis G. (2015). Evaluation of lunar regolith geopolymer binder as a radioactive shielding material for space exploration applications. Adv. Space Res..

[30] Davis G., Montes C., Eklund S. (2017). Preparation of lunar regolith based geopolymer cement under heat and vacuum. Adv. Space Res..

[31] Lu W., Shi Y., Xue X., Li H. (2025). Mechanical properties of short-age CQU-1 lunar regolith simulant geopolymer for in-Situ resource utilization. Constr. Build. Mater..

[32] Zhan J.H., Xue X.Y., Hua J.M., Huang L.P. (2025). Effects of curing temperature on early-age mechanical property and microstructure of lunar regolith simulant geopolymer. Case Stud. Constr. Mater..

[33] Hua J.M., Xiao C., Xue X.Y., Huang L.P. (2025). Experimental study on physical and mechanical properties of simulated lunar golith hardened by radiation sintering. J. Civ. Environ. Eng..

[34] Xue X.Y., Hua J., Xiao C., Huang L.P. (2024). Prospects for Intelligent Construction Technology for Lunar Base in Extreme Environments. Sci. Technol. Foresight.

[35] Zhan J.H., Yi H.L., Wang N., Wang F., Li S., Hua J.M., Xue X.Y. (2025). Effect of Basalt Fiber Content on Mechanical Properties of Lunar Regolith Simulant Geopolymer Under Static Loading. Materials.

[36] Kanamori H. (1998). Properties of Lunar Soil Simulant Manufactured in Japan. Space.

[37] (2021). Test Method of Cement Mortar Strength (ISO Method).

[38] (2016). Methods of Testing Cement Part 1: Determination of Strength.

[39] (2010). Code for Design of Concrete Structures.

[40] Yang S.Z., Liu B.D., Li F. (2022). Estimations of the elastic moduli of concrete at different temperatures and humidities by mesomechanics methods. Case Stud. Constr. Mater..

[41] Aili A., Vandamme M., Torrenti J.M., Masson B., Sanahuja J. (2016). Time evolutions of non-aging viscoelastic Poisson’s ratio of concrete and implications for creep of C-S-H. Cem. Concr. Res..

[42] Guo Z.H., Zhang X.Q. (1987). Investigation of Complete Stress-Deformation Curves for Concrete in Tension. ACI Mater. J..

[43] Hognestad E., Hanson N.W., McHenry D. (1955). Concrete stress distribution in ultimate strength design. J. Acids.

[44] Carreira D.J., Chu K.H. (1985). Stress-strain relationship for plain concrete in compression. J. Am. Concr. Inst..

[45] Mukheef D., Shaimaa H., Wisam S., Sultan H. (2025). Representation of stress strain curve for compression behavior of concrete using trigonometric function model. J. Build. Pathol. Rehabil..

